# The feasibility and outcome of a community-based primary prevention program for cardiovascular disease in the 21st century

**DOI:** 10.1080/02813432.2021.1913893

**Published:** 2021-06-07

**Authors:** Susanna M. Kuneinen, Johan G. Eriksson, Hannu Kautiainen, Mikael O. Ekblad, Päivi E. Korhonen

**Affiliations:** a Department of General Practice, Turku University and Turku University Hospital, Turku, Finland; b Central Satakunta Health Federation of Municipalities, Harjavalta, Finland; c Folkhälsan Research Center, Helsinki, Finland; d Department of General Practice and Primary Health Care, University of Helsinki and Helsinki University Hospital, Helsinki, Finland; e Department of Obstetrics and Gynecology, Yong Loo Lin School of Medicine, National University of Singapore, Singapore, Singapore; f Agency for Science, Technology and Research (A*STAR), Singapore Institute for Clinical Sciences (SICS), Singapore, Singapore; g Unit of Primary Health Care, Kuopio University Hospital, Kuopio, Finland

**Keywords:** Cardiovascular disease, mortality, primary prevention, screening, general practice

## Abstract

**Objective:**

There is no evidence that systematic screening and risk factor modification in an unselected, asymptomatic population will reduce cardiovascular disease (CVD) mortality. This study aimed to evaluate the effectiveness of a primary care CVD prevention program on mortality during a 13-year follow-up.

**Design:**

A risk factor survey was sent, followed by a nurse-led lifestyle counselling to respondents with at least one CVD risk factor, and a general practitioner’s (GP) appointment for high-risk persons. Screening and interventions were performed during 2005–2006.

**Setting:**

A public health care centre in the town of Harjavalta, Finland.

**Subjects:**

All home-dwelling 45–70-year old inhabitants without manifested CVD or diabetes.

**Main outcome measures:**

All-cause and CVD mortality.

**Results:**

Altogether 74% (2121/2856) inhabitants responded to the invitation. The intervention was received by 1465 individuals (52% of the invited population): 398 risk persons had an appointment with a nurse, followed by an appointment with a GP for 1067 high-risk persons. During the follow-up, 370 persons died. Mortality among the non-respondents was twofold compared to the participants’. In subjects who received the intervention, the age- and gender-adjusted hazard ratio for all-cause mortality was 0.44 (95% CI: 0.36 to 0.54) compared to the subjects who did not receive the intervention.

**Conclusions:**

Reducing mortality is possible in a primary care setting by raising health awareness in the community with screening, by targeted lifestyle counselling and evidence-based preventive medication for persons at high risk for CVD. Subjects not willing to participate in health surveys have the worst prognosis.

**Key Points**
- Previously, there is no evidence that systematic screening and risk factor modification in an unselected, asymptomatic population will reduce cardiovascular disease (CVD) mortality.
- With a stepwise screening program it is possible to scale the magnitude of CVD prevention in the community.
- Reducing mortality in a community is possible by screening, targeted lifestyle counselling, and by evidence-based preventive medication for high-risk persons.
- Subjects not willing to participate in health surveys have the worst prognosis.

## Introduction

Cardiovascular disease (CVD) is globally the leading cause of disability and mortality [1]. Over 70% of CVD cases can be attributed to few modifiable risk factors such as smoking, hypertension, obesity, sedentary lifestyle, and unhealthy diet [2]. Yet, there is no evidence from randomized controlled trials (RCTs) that systematic screening and risk factor modification in an unselected, asymptomatic population will reduce total or cause-specific CVD mortality [3].

At the population level, prevention programs have been successful in reducing CVD mortality rates. Since the North Karelia Project – the first large community-based CVD prevention program in the world – was launched in 1972, premature coronary heart disease (CHD) mortality in Eastern Finland has decreased more than 80% over a period of 40 years [4]. Even between the years 2002–2012, i.e. in the high era of modern cardiological treatments and interventions, two-thirds of the observed decline in CHD mortality could be explained by reductions in population levels of cholesterol, systolic blood pressure, and smoking prevalence [4]. Similarly in Sweden and Iceland, the largest effects on coronary mortality have come from primary prevention, attributable to reductions in major cardiovascular risk factors in the population [5,6].

Implementation of these successful population-based strategies aiming at CVD prevention is challenging in primary care. General practitioners (GPs) traditionally interact with one patient at a time, perform individual risk assessment, and primarily manage high-risk subjects. Although individuals at high risk for CVD gain the most from preventive measures, the majority of CVD mortality occurs in low or intermediate-risk individuals simply because they form the majority of the population [7]. It has been suggested that successful primary prevention of CVD requires both a population strategy and a high-risk strategy [8].

In Finland, municipalities are responsible for organizing primary health care for their residents. The contents of services are defined by law and they include monitoring the health of the population and health counselling. This article describes a screening and intervention program carried out in the semirural town of Harjavalta (7673 inhabitants in the year 2005) in southwestern Finland. This Harmonica Project (Harjavalta Risk Monitoring for Cardiovascular Disease) used a combined population and high-risk strategy aiming to prevent or postpone CVD in the community [9]. We now report the findings on mortality after 13 years of follow-up. We sought to investigate if the risk of death varied between the identified study population groups, and to compare mortality between the Harjavalta cohort and the general population of Finland over the same period of time.

## Methods

### Subjects

Men and women aged 45–70 years living in Harjavalta (3002 inhabitants of the age group in 2005) were invited to participate in the Harmonica Project. Institutionalized persons and people with previously diagnosed diabetes or CVD who already had a regular follow-up in the health care centre (*n* = 146) were not invited. Screening and interventions were performed from August 2005 to October 2006.

A cardiovascular risk factor survey, a tape for the measurement of waist circumference (WC), and a type 2 diabetes (T2D) risk assessment questionnaire (FINDRISC, Finnish Diabetes Risk Score, available from www.diabetes.fi/english) [10] were mailed to every eligible inhabitant (*n* = 2856, i.e. 95% of the eligible population). The subjects were asked to measure their WC at the level of umbilicus, to report the latest measured blood pressure (BP), their use of antihypertensive medication, their history of gestational diabetes or hypertension, and first-degree family history of CHD, myocardial infarction, or stroke. The subjects were asked to mail the risk factor survey back to the health care centre if they were willing to participate in the project. Participation and all the tests included were free of charge for the subjects. Response rate was 74%. Thirteen respondents were excluded because of a missing risk factor survey, resulting in an analytical cohort of 2843 persons.

The respondents with at least one reported CVD risk factor (listed below) were invited for laboratory tests and an appointment with a trained public health nurse within two months.

- Waist circumference ≥80 cm in women, ≥94 cm in men [11]
- Finnish Diabetes Risk Score ≥12, estimated at least 1 in 6 will develop type 2 diabetes within 10 years [10]
- Most recently measured blood pressure ≥140 mmHg systolic or ≥90 mmHg diastolic
- Use of antihypertensive medication
- History of gestational diabetes or hypertension
- First-degree family history of premature cardiovascular disease.

### Appointment with the public health nurse

Measurements of BP with a mercury sphygmomanometer, height, weight, and waist circumference were made by a trained nurse. The measurement procedures have been described in detail previously [9].

If the mean systolic BP was ≥140 mmHg or the mean diastolic BP ≥90 mmHg, subjects were instructed to use an automatic BP monitor (Omron M4-1, the Netherlands), which was lent to them to execute a one week of home BP monitoring [9].

Body mass index (BMI) was calculated as weight (kg) divided by the square of height (m^2^). Metabolic syndrome (MetS) was defined according to the criteria of the International Diabetes Federation (IDF) [11].

The Systematic Coronary Risk Evaluation (SCORE) system was used to estimate the 10-year risk of CVD death in subjects aged 45 to 65 years [12].

Blood was drawn after at least 12 h of fasting. Total cholesterol, HDL cholesterol and triglycerides were measured enzymatically (Olympus® AU640, Japan). LDL cholesterol was calculated according to the Friedewald’s formula [13]. A 2-hour oral glucose tolerance test (OGTT) was performed by measuring fasting plasma glucose and a 2-hour plasma glucose after ingestion of a glucose load of 75 g anhydrous glucose dissolved in water. Glucose values were measured from capillary whole blood with HemoCue^®^ Glucose 201+ system (Ängelholm, Sweden). The analyzer converts the result from capillary whole blood to plasma glucose (conversion factor 1.11).

Participants completed self-administrative questionnaires at the clinic while waiting for the nurse’s appointment. These included dietary habits, leisure-time physical activity (LTPA), sociodemographic factors, educational attainment, smoking status (current/no), Alcohol Use Disorders Identification Test (AUDIT) [14], and Beck’s Depression Inventory (BDI) [15]. The first question of Short-Form Health Survey (SF-36) [16] was used to assess self-rated health. A BDI score ≥10 was used as definition of increased depressive symptoms [17]. LTPA level was categorized as low (LTPA for ≥30 min at a time for maximum of three times a week), moderate (LTPA for ≥30 min at a time for four to five times a week), and high (LTPA ≥30 min at a time for six or more times a week).

The nurse explained the test results and gave verbal and written lifestyle information to all subjects individually. The main goal of the lifestyle counselling was to reduce intake of saturated fat in the diet and to perform LTPA at least 30 min per day or four hours per week. Overweight and obese persons (BMI ≥25.0 kg/m^2^) were encouraged to weight reduction of at least 5%. Every subject had his/her test results written down in a notebook along with target values. The nurse’s appointment took approximately an hour including examinations and lifestyle counselling.

Those participants who were regarded as having high CVD risk (listed below) were offered to have an appointment with the GP of the project.

- Hypertension: the subject was already taking antihypertensive therapy, OR the mean of home BP monitoring was ≥135 mmHg systolic or ≥85 mmHg diastolic
- Newly diagnosed diabetes [18]^:^ fasting plasma glucose ≥7.0 mmol/l, OR 2-hour plasma glucose concentration ≥12.2 mmol/l
- Impaired glucose tolerance: 2-hour plasma glucose concentration 8.9-12.1 mmol/l
- Metabolic syndrome [11]: waist circumference ≥80 cm in women or ≥94 cm in men AND any two of the following:
  - nurse-measured BP ≥130 systolic or ≥85 mmHg diastolic or antihypertensive drug therapy
  - fasting plasma glucose ≥5.6 mmol/l or newly diagnosed type 2 diabetes
  - fasting plasma triglycerides ≥1.7 mmol/l or specific drug therapy
  - fasting plasma HDL-C <1.29 mmol/l in women or <1.03 mmol/l in men or specific drug therapy
- Body mass index ≥30.0 kg/m^2^
- Ten year risk for cardiovascular death now or extrapolated to 60 years of age ≥5% [12]

### Appointment with the general practitioner

The GP’s appointment was scheduled within 2–4 months after the nurse’s appointment. At that time, plasma lipids and fasting plasma glucose were retested. An ECG and laboratory tests were collected. The GP examined the patients and provided lifestyle counselling. According to the national Finnish guidelines of that time, antihypertensive medication was initiated if systolic BP was ≥160 mmHg or diastolic ≥100 mmHg, or target organ damage was diagnosed. Ongoing antihypertensive medication was intensified if systolic BP was ≥140 mmHg or diastolic ≥85 mmHg (≥80 mmHg in patients with diabetes). If the 10 year risk for developing a fatal cardiovascular event now or extrapolated to the age of 60 years was ≥5% [12], preventive medication – an antihypertensive drug, a lipid lowering agent or low dose aspirin – was initiated.

### Formation of the study groups

**Figure 1.**
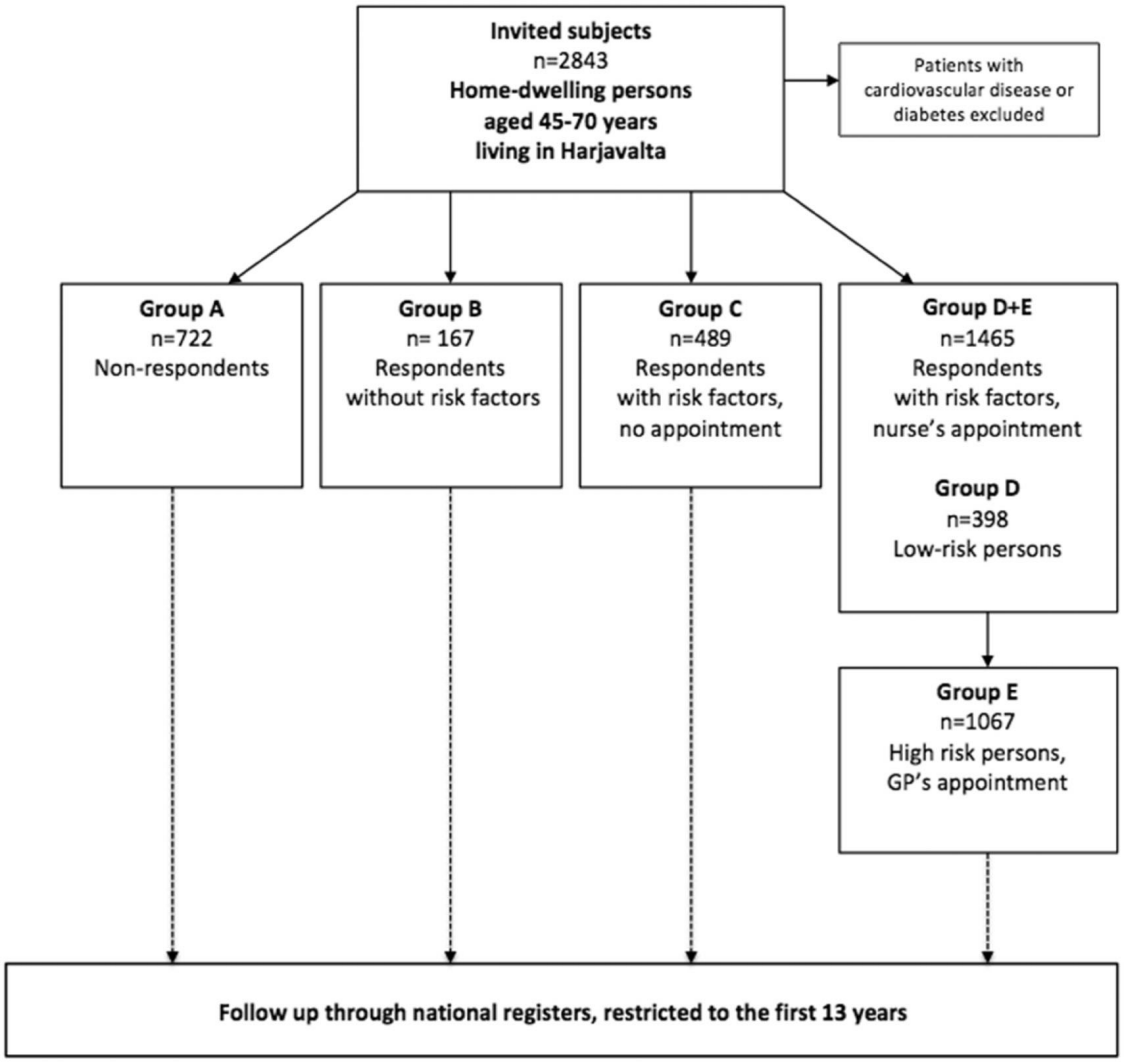
The design of the Harmonica Project and the groups studied.

The subjects were divided into groups (A-E) according to their response to the invitation to participate, the findings in the risk factor survey, and the findings based on the measurements made at the nurse’s appointment (Figure 1).

### Ethical approval

The study protocol and consent forms were reviewed and approved by the ethics committee of Satakunta hospital district. All participants provided written informed consent for the project and subsequent medical research.

### Mortality

Data on mortality was obtained from the national statistical authority, Statistics Finland. Causes of death were classified according to International Classification of Diseases and Related Health Problems, 10^th^ Revision (ICD-10): diseases of the circulatory system (I00-I99), and deaths from all causes. For each person, the date of the invitation to the Harmonica project was the start date of the observational period. Follow-up time of mortality ended on December 31^st^, 2018.

### Statistical analyses

Data are presented as means with standard deviation (SD) or as counts with percentages. Statistical comparisons between groups were made using t test, analysis of variance (ANOVA) and Pearson's chi‐square test. Survival curves were constructed according to the Kaplan-Meier method and adjusted using Cox regression model. Cox proportional hazards model was used to calculate the adjusted hazard ratios (HR) with 95 per cent confidence intervals (95% CI). The ratio of observed to expected number of deaths, the standardized mortality ratio (SMR) for all-cause and CVD deaths, was calculated using subject-years methods with 95% confidence intervals, assuming a Poisson distribution. The expected number of deaths was calculated on the basis of sex-, age- and calendar-period-specific mortality rates in the Finnish population (Official Statistics of Finland). Hommel’s adjustment was applied to correct levels of significance for multiple testing of group differences in SMR. The normality of variables was evaluated using graphically and the Shapiro–Wilk W test. Stata 16.0 (StataCorp LP, College Station, TX, USA) was used for the analysis. Stata version 16.1 (StataCorp, College Station, TX) was used for all statistical analyses.

## Results

### Study population

The study population consisted of 2843 home-dwelling 45-70-year old subjects who had no manifested CVD or diabetes. Their mean age was 57 years (SD 7), 54% being females.

There were 722 (25%) subjects who did not respond to the survey (Group A). Of the 2121 respondents, 167 (8%) reported no risk factors (Group B), whereas 1954 (92%, Groups C–E) reported at least one CVD risk factor in the risk factor survey. The most common risk factor was elevated WC, followed by elevated blood pressure, increased risk for T2D, and family history of CVD (Table 1).

**Table 1.** Baseline characteristics of the study participants.

	Group A *n* = 722	Group B *n* = 167	Group C *n* = 489	Group D *n* = 398	Group E *n* = 1067	*p* Value (Multiple comparison^a^)
Women, *n* (%)	386 (53)	92 (55)	198 (40)	135 (34)	505 (47)	<0.001
Age, mean (SD)	56 (7)	56 (7)	57 (7)	56 (7)	58 (7)	<0.001
Reported risk factors, *n* (%)						
Waist circumference	..	0 (0%)	304 (62)	358 (90)	1005 (94)	<0.001^b^
Hypertension	..	0 (0%)	230 (47)	139 (35)	692 (65)	<0.001^b^
FINDRISC score ≥12	..	0 (0%)	102 (21)	121 (30)	547 (51)	<0.001^b^
Gestational diabetes or hypertension^a^	..	0 (0%)	44 (15)	35 (13)	97 (17)	0.33^b^
Family history of CVD	..	0 (0%)	281 (57)	276 (69)	687 (64)	<0.001^b^
Measured risk factors, mean (SD)						
BMI, kg/m^2^	..	..	..	25.6 (2.5)	30.5 (28.1)	<0.001
Obesity (BM*I* ≥ 30.0), *n* (%)				13 (3)	448 (42)	..
Waist circumference, cm	..	..	..			
Women	..	..	..	83 (7)	94 (13)	<0.001
Men	..	..	.	94 (8)	102 (11)	<0.001
Blood pressure, mmHg						
Systolic	..	..	.	129 (15)	144 (18)	<0.001
Diastolic				80 (9)	87 (10)	<0.001
Plasma glucose, mmol/l						
Fasting	..	..	..	5.3 (0.6)	5.7 (1.1)	<0.001
2-hour postload	..	..	..	6.5 (1.4)	7.7 (2.5)	<0.001
Plasma lipids, mmol/l	..	..	..			
Total cholesterol	..	..	..	5.3 (0.9)	5.4 (1.0)	0.046
LDL cholesterol	..	..	..	3.1 (0.8)	3.2 (0.9)	0.005
HDL cholesterol	..	..	..			
Women	..	..	..	1.83 (0.43)	1.62 (0.41)	<0.001
Men	..	..	..	1.51 (0.36)	1.39 (0.39)	0.003
Triglycerides	..	..	..	1.10 (0.52)	1.48 (0.77)	<0.001
Health behaviours						
Current smoker, *n* (%)	..	..	..	55 (15)	206 (19)	0.039
AUDIT score, mean (SD)	..	..	..	4.1 (4.2)	5.0 (5.2)	0.001
LTPA, *n* (%)	..	..	..			<0.001
Low	..	..	..	43 (11)	197 (19)	
Moderate	..	..	..	197 (52)	516 (51)	
High	..	..	..	141 (37)	306 (30)	
Psychosocial factors						
Educational attainment years, mean (SD)	..	..	..	11.0 (2.8)	10.3 (2.6)	<0.001
Depressive symptoms, *n* (%) (BD*I* ≥ 10)	..	..	..	64 (16)	200 (19)	0.24
Poor self-rated health, *n* (%)	..	..	..	102 (27)	414 (41)	<0.001
Person-years followed up	8607	2010	6093	5162	13555	..
Total number of deaths at follow-up, *n* (%)	166 (23)	23 (14)	82 (17)	29 (7)	122 (11)	**..**
Standardized mortality ratio (95% CI)						
All-cause mortality	1.82 (1.57–2.12)	0.96 (0.64–1.45)	1.24 (1.00–1.54)	0.58 (0.40–0.84)	0.71 (0.59–0.84)	<0.001 [A/B, A/C, A/D, A/E, C/D, C/E]^a^
CVD mortality	1.61 (1.19–2.14)	0.51 (0.14–1.30)	0.89 (0.54–1.39)	0.32 (0.11–0.76)	0.50 (0.34–0.72)	<0.001 [A/B, A/C, A/D, A/E, C/D, C/E]^a^

^a^Hommel’s multiple comparison procedure was used to correct significance levels for post hoc testing (*p* < 0.05).

^b^Comparison between the Groups C–E.

FINDRISC: Finnish Diabetes Risk Score; CVD: cardiovascular disease; BMI: Body mass index; OGTT: oral glucose tolerance test; LDL: low-density lipoprotein; HDL: high-density lipoprotein; AUDIT: Alcohol Use Disorders Identification Test; LTPA: leisure-time physical activity.

There were 489 risk persons (23% of the respondents) who did not want to attend the appointment with a nurse or a GP (Group C). The intervention was received by 1465 individuals (52% of the invited population), 398 low-risk persons (Group D) had an appointment with a nurse, and 1067 high-risk persons (Group E) had appointments both with a nurse and with a GP. In persons regarded as high-risk (Group E, 50% of the respondents), all measured CVD risk factor levels were on average higher than in those regarded as low-risk persons (Group D). High-risk subjects also had higher mean AUDIT scores, performed less LTPA, and were less educated than low-risk subjects. Although high-risk subjects had no more depressive symptoms, their self-rated health was more often poor than that of low-risk persons (Table 1).

Of the high-risk persons (*n* = 1067, Group E), 483 (45%) had ongoing antihypertensive medication, but only 166 (34%) reached the treatment target <140/90 mmHg. Antihypertensive medication was initiated for 72 (7%) newly diagnosed hypertensive subjects. Lipid lowering medication was ongoing in 166 (16%) persons and new lipid lowering medication was prescribed for 180 (17%). Another visit with the GP was scheduled for 360 (34%) high-risk persons.

### Mortality in the study groups

**Figure 2.**
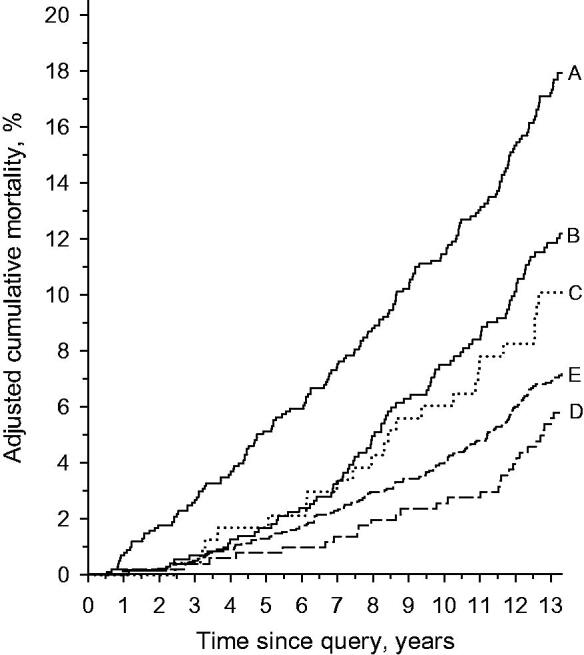
Cumulative all-cause mortality curves adjusted for age and gender.

Unadjusted cumulative all-cause mortality over 13 years were as follows: 22.2% (95% CI: 19.3 to 24.4) in Group A, 13.8 (95% CI: 9 to 20.0) in Group B, 16.4 (95% CI: 13.4 to 20.1) in Group C, 7.0 (95% CI: 5.0 to 10.0) in Group D, and 11.1 (95% CI: 9.3 to 13.1) in Group E. The age- and gender-adjusted cumulative all-cause mortality is illustrated in Figure 2. In subjects who received the intervention (Groups D + E), the age- and gender-adjusted hazard ratio (HR) for all-cause mortality was 0.44 (95% CI: 0.36 to 0.54) compared to the subjects who did not receive the intervention (Groups A + B + C). In low-risk subjects (Group D) the adjusted HR for all-cause mortality was 0.79 (95% CI: 0.53 to 1.19, *p* = 0.27) compared to the high-risk subjects (Group E).

**Figure 3.**
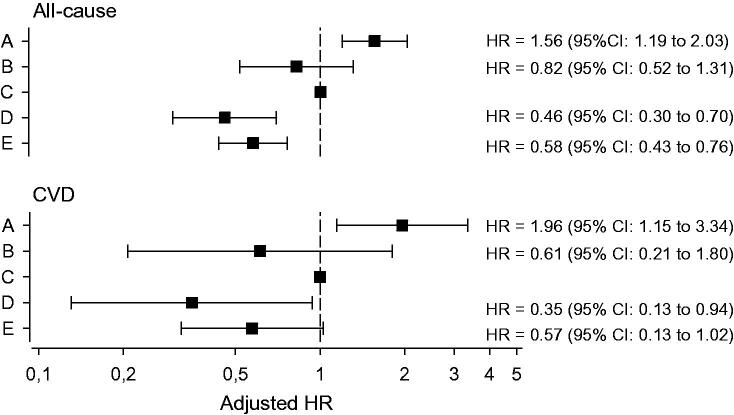
Hazard ratios (HRs) for all-cause and cardiovascular disease (CVD) mortality in the study groups with Group C as the reference. Adjustments were made for age and gender. Whiskers show 95% confidence intervals.

Figure 3 shows the age- and gender-adjusted HRs for all-cause mortality and CVD mortality with the Group C (risk persons who showed no interest on intervention) set as the reference.

When adjusted for age, gender, and risk factors reported in the risk factor survey, and Group C set as the reference, HRs for all-cause mortality and CVD mortality were, respectively in Group D 0.35 (95% CI: 0.21 to 0.57) and 0.24 (95% CI: 0.08 to 0.74), and in Group E 0.48 (95% CI: 0.35 to 0.66) and 0.41 (95% CI: 0.21 to 0.79).

### Mortality compared to the finnish general population during the follow-up

**Figure 4.**
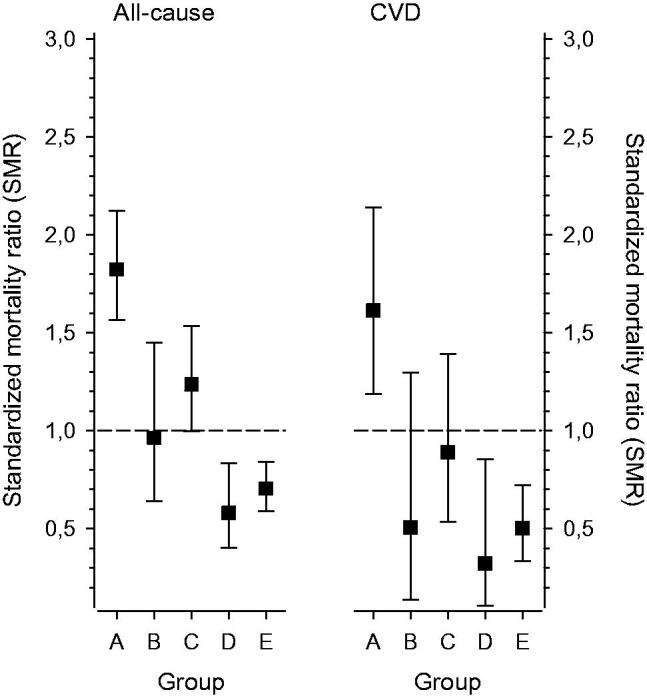
Standardized mortality ratio (SMR) for all-cause and cardiovascular disease (CVD) deaths in the study groups during the follow-up period. Whiskers show 95% confidence intervals.

Figure 4 displays SMR for all-cause and CVD deaths in the study groups during the 13-year follow-up. Those inhabitants of Harjavalta who showed no interest in the survey (Group A) had increased SMR and those who received the intervention (Groups D + E) lower SMR compared to the mortality rate throughout Finland over the same period (Table 1).

## Discussion

This community-based CVD prevention program engaged 74% of the invited middle-aged, apparently healthy population. The two-step screening-method managed to limit the number of GP’s appointments for those 50% who might benefit from preventive medication. In the CVD risk-population, lifestyle counselling accompanied by preventive medication if indicated, was associated with a 13-year mortality rate comparable to the respondents with no risk factors at baseline. Total and CVD mortality rates among the 25% who showed no interest to participate in the screening, was twofold higher than among the respondents.

Even though the Harmonica Project was carried out in a small urban community, the total number of hard end points, i.e. all-cause mortality, was sufficient to show a positive effect of screening and intervention. Because of the non-RCT design of the study, caution is warranted in the interpretation of the results. We do not have sufficient information on the health of the non-respondents (Group A), we only know that they were 45–70-years old and had no verified CVD or T2D in medical health records. Thus, we cannot rule out the effect of non-response bias and self-selection bias. Moreover, non-respondents of preventive programs often have a less healthy lifestyle and may have lower socioeconomic position than active participants [19,20].

Further, we do not know whether the respondents with risk factors but not willing to participate in the intervention (Group C) were at low or at high CVD risk. Thus, although we hypothesize that the lower mortality risk in Group D and E (low-risk and high-risk persons) than in Group C stems from the multifactorial intervention provided, it may also be partly due to the different risk level status at baseline. However, relative mortality among the risk persons who did receive the intervention in the public health centre, was significantly lower compared to the Finnish general population.

The study was executed at a time when the IDF criteria for the metabolic syndrome (MetS) received large attention and criticism, and especially the strict cut-off value for WC might have overestimated the number of individuals at risk. However, early prevention of overweight and obesity is recommended by the WHO [21] since the contemporary population data shows sustained weight gain in adults [22] also in Finland [23]. The so called high-risk group referred to the GP’s appointment was chosen from the subjects who might benefit from repeated lifestyle counselling and perhaps from preventive medication.

In the present study, Group B with no risk factors was relatively small indicated by the wide confidence intervals overlapping with Group C. Moreover, separating people into different risk groups is always somewhat artificial since the risk factors for CVD form a continuum. However, dichotomization of the continuous variables makes clinical decisions easier [7]. It is probable that many cardiometabolically healthy subjects at baseline became CVD risk persons during the 13-year follow up.

The intervention used in the Harmonica Project was typical for general practice. At the same appointment, several issues were taken into account and personalized risk assessment was used to select the intensity of the intervention. The public health nurses and the GP who examined the risk persons and gave counselling, were permanent staff of the public health care centre. Thus, the secured continuity of care was one factor contributing to the results [24]. The public health care centre also started co-operation with the community recreational facilities to provide opportunities to physical activities for all inhabitants of the community. Nurse and GP driven public lectures were regularly arranged, and news about healthy lifestyle were written in the local newspaper. Thus, the Harmonica Project managed to raise community’s health awareness, which is perhaps too often forgotten in health care. It is easier for an individual to adopt a healthier lifestyle when other members of the community share the effort [7].

A Cochrane review on multiple risk factor intervention for primary prevention of CHD included 39 RCTs, but only ten reported total or CHD mortality as an outcome. Overall, no effect on mortality was found, except in high-risk hypertensive populations [25]. In the Harmonica Project, hypertension was diagnosed in 52% of the high-risk subjects thus being the most noteworthy CVD risk factor in the community. Recent reports from the large randomized population-based intervention studies with long follow-up times (10 and 30 years), the Inter99 [26] and the Danish MONICA study [27], evaluated the effect of repeated screening and lifestyle counselling, and found no effect on CVD morbidity or death. It must be noted that our study design is not comparable with the aforementioned RCTs. We did not perform randomization of the inhabitants in order to preserve the communal effect. Also, in our study the intervention was provided by familiar nurses and GP at the local health care center, thus continuity of care was secured. These factors might play a significant role in the success of the intervention.

The majority of previous large community-based studies of CVD screening and intervention reporting decreased mortality [4,28] have been initiated in the 1970s or 1980s when risk factor burden of the general population was considerably higher than in the twenty first century. Therefore, it is probably more difficult for present-day studies to demonstrate an effectiveness of primary prevention. The positive effect of the community in preventing CVDs has been recognized previously. Lee *et al* reported that participation in CVD health screening and educational counselling or treatment referral was associated with lower rates of CVD and all-cause mortality [29]. A recent Japanese study showed that individual and area-level interest for health screening reduces mortality rates. The results suggest that people’s interest in health screening might reflect the community’s overall health consciousness [30].

The risk factor burden of the general population is largely hidden from health care, unless risk factors are not actively screened for [7]. Special efforts are needed to stimulate interest in the non-respondents of health surveys. A risk factor survey might work as a health indicator in itself and also as a motivational factor for participation. Subjects not willing to participate probably constitute a significant bias to clinical trials. Targeted screening, lifestyle counselling and evidence-based preventive medication for persons at risk for CVD seem to be effective in reducing mortality in primary health care setting.
